# Psychometric Assessment of the Rat Grimace Scale and Development of an Analgesic Intervention Score

**DOI:** 10.1371/journal.pone.0097882

**Published:** 2014-05-16

**Authors:** Vanessa Oliver, Debbie De Rantere, Rheanne Ritchie, Jessica Chisholm, Kent G. Hecker, Daniel S. J. Pang

**Affiliations:** 1 Department of Veterinary Clinical and Diagnostic Sciences, Faculty of Veterinary Medicine, University of Calgary, Calgary, AB, Canada; 2 Department of Zoology, Faculty of Biological Sciences, University of Calgary, Calgary, AB, Canada; 3 Hotchkiss Brain Institute, University of Calgary, Calgary, AB, Canada; Brock University, Canada

## Abstract

Our limited ability to assess spontaneous pain in rodent models of painful human conditions may be associated with a translational failure of promising analgesic compounds in to clinical use. If measurement of spontaneous pain behaviours can be used to generate an analgesic intervention score their use could expand to guide the use of analgesics, as mandated by regulatory bodies and ethical and welfare obligations. One such measure of spontaneous pain, the Rat Grimace Scale (RGS), has recently been described and shown to exhibit reliability. However, reliability of measurement scores is context and content specific, and further testing required to assess translation to a heterogenous setting (different model, raters, environment). The objectives of this study were to perform reliability testing with the Rat Grimace Scale in a heterogenous setting and generate an analgesic intervention score for its use. In a randomised, blinded study, sixteen adult female rats received one of three analgesia treatments (0.05 mg/kg buprenorphine subcutaneously, 1 mg/kg meloxicam subcutaneously, 0.2 mg/kg oral buprenorphine in jelly) peri-operatively (telemetry unit implantation surgery). Rats were video-recorded (before, 1–6 and 12 hours post-operatively) and images collected for independent scoring by three blinded raters using the RGS, and five experts based on “pain/no pain” assessment. Scores were used to calculate inter- and intra-rater reliability with an intraclass correlation coefficient and generate an analgesic intervention score with receiver operating characteristic curve analysis. The RGS scores showed very good inter- and intra-rater reliability (0.85 [0.78–0.90 95% CI] and 0.83 [0.76–0.89], respectively). An analgesic intervention threshold of greater than 0.67 was determined. These data demonstrate that the RGS is a useful tool which can be successfully employed in a heterogenous setting, and has the potential to guide analgesic intervention.

## Introduction

The recognition and assessment of spontaneous behaviours associated with pain in laboratory species has been identified as an area requiring further investigation in biomedical research and veterinary medicine [1]–[5]. This need has emerged from the poor predictive ability of animal models of pain [1], [3], the ethical and regulatory obligations of providing appropriate analgesia [6]–[9] and the fundamental requirement to understand and assess the efficacy of analgesic agents in animals [2], [10].

Until recently, pain scales used in animals, many of which have been adopted from medicine have not undergone rigorous assessment for their reliability and validity [11]–[14]. As appreciation for the importance of scale assessment for validity and reliability using psychometric methods grows, pain scales are being developed with these principles in multiple species including dogs, cats, mice and rats [4], [5], [14]–[19].

Most recently, based on apparent evolutionary conservation of facial expressions and successful employment of facial action coding in non-verbal humans, facial expression scoring has been applied in rats, mice and rabbits [15], [16], [20], [21]. The practical utility and widespread adoption of these scales require evidence of the validity (does a scale measure what it claims to measure) and reliability (measurement error associated with a scale) of these scores. Aspects of validity (construct and content) have been addressed for the Rat (RGS), Mouse (MGS) and Rabbit Grimace Scales [10], [15], [16], [21], [22].

Reliability of pain scale scores can be assessed using internal consistency, inter- and intra-rater reliability. Internal consistency reflects the degree to which scale items are inter-related, while inter- and intra-reliability quantify the ability of a scale to return similar measures between different raters and the same rater at different times, respectively. A single reliability study is insufficient to allow generalisation to a heterogenous setting (different study populations, environments and raters), and Sotocinal et al. (2011), in their initial validation of the RGS, encouraged others to assess their scale [16]. Through repeated use and publication, this will eventually allow reliability generalisation, a concept similar to meta-analysis, as applied to measurement scales [23].

Identification of an analgesic intervention score for a pain scale dramatically increases its utility, expanding its use from an observational research tool to one facilitating decision making and intervention [24]. This moves towards fulfilling the aforementioned goals of providing appropriate analgesia and assessment of the efficacy of analgesic agents. At a fundamental level, identification of an analgesic intervention score allows personnel involved in animal work to fulfil a “duty of care” towards non-verbal subjects [14].

The aims of this study were to further assess reliability of the RGS and identify an analgesic intervention score.

## Materials and Methods

Adult female Sprague-Dawley rats (284–420 g), obtained from surplus stock at the University of Calgary, were scheduled for surgical implantation of a telemetric radio-transmitter device (4ET-S2 Radio Transmitter, Data Sciences International, Saint Paul, MN, USA) as part of an unrelated study. Animals were randomised to receive one of three analgesia treatments peri-operatively or enter a sham treatment group. Animals were maintained in a 12 hr–12 hr light-dark cycle (lights on at 0700) and housed in pairs or groups of three in micro-filter cages (48×27×20 cm [Ancare Corp., Worcester, MA, USA]) and were provided both fresh water and food (Prolab 2500 Rodent 5p14, Lab diet, PMI Nutrition International, St Louis, MO, USA) *ad libitium*. Plastic tubing (PVC Pipe, provided by the Health Science Animal Resource Centre, Calgary, Alberta CA) wood shavings (Aspen chip, NEPCO, Warrensburg, NY, USA) and shredded paper were provided for nesting and cage enrichment. All surgeries were completed between 1000 h and 1600 h.

All animals received the following perioperative protocol to facilitate surgery: induction of general anaesthesia with isoflurane (Isoflurane USP, Pharmaceutical Partners of Canada Inc., Richmond Hill, ON, Canada) carried in oxygen, provision of antibiosis (enrofloxacin [Baytril 50 mg/mL, Bayer, Toronto, ON, Canada] 5 mg/kg subcutaneously [SC]), an incisional line block (2 mg lidocaine [Lidocaine HCl 2%, Wyeth Animal Health, Guelph, ON, Canada] diluted in 0.9 mL NaCl distributed SC at the incision sites) and fluids (4.0 mL 0.9% NaCl, SC [Baxter Corporation, Mississauga, ON, Canada]). Antibiotic, line block, analgesia and fluid injections were administered after loss of righting reflex occurred. Surgery to implant the telemetric device began following confirmation of loss of pedal withdrawal reflex. The radio-transmitter and battery units were located in a latero-dorsal subcutaneous pocket. From these, leads for electroencephalography, electromyography and electrocardiography were tunnelled subcutaneously to the dorsal surface of the skull, trapezius muscles and pectoral muscles, respectively. Two separate incisions, over the skull and midway between the axillae and sternum, were made to facilitate lead placement. At the end of surgery, a further 4 mL of 0.9% NaCl was administered SC. Animals were housed singly following instrumentation.

The three analgesic treatment groups consisted of buprenorphine (0.05 mg/kg SC, pre-operatively and at 6, 12 and 24 hours post-operatively [Vetergesic, 0.3 mg/mL Champion Alstoe Animal Health Inc., Whitby, ON, Canada]), meloxicam (1 mg/kg SC, pre-operatively and 24 hours post-operatively [Metacam 5 mg/mL, Boehringer Ingelheim, Burlington, ON, Canada), and oral buprenorphine (0.05 mg/kg SC pre-operatively, followed by 0.2 mg/kg in grape jelly (Grape Jell-O, Kraft Foods, North York, Ontario, CA) offered at 6, 12 and 24 hours post-operatively). Rescue analgesia, consisting of buprenorphine (0.05 mg/kg SC), was administered if an observer felt an animal was in pain based on clinical impression.

Animals randomised to the sham treatment group received the same handling and treatment (incisional block, fluid, antibiotic) as the the instrumented groups with the exception of surgery itself. All animals in the sham treatment group received the same analgesia protocol (buprenorphine and meloxicam at the dosing intervals described above) and were maintained under general anaesthesia for the same average duration as the instrumented group.

Video-recording, with each animal in its home cage (enrichment removed during recording), was performed at the following time points post-operatively: 1, 2, 3, 4, 5, 6, 12 hrs. Video-recording was performed prior to analgesic administration when the recording and analgesia schedules coincided. Each recording period lasted 30 minutes and video was recorded from two cameras [Panasonic HC-V720P/PC, Panasonic Canada Inc., Mississauga, ON, Canada] simultaneously, placed orthogonally. Baseline video-recordings were performed at least 24 hours prior to surgery following the same 30 minute recording protocol. No personnel were present in the room during the recording period. Video files were downloaded to a computer and image selection for application of the Rat Grimace Scale was as described by Sotocinal et al. (2011) [16]. Briefly, up to ten still images were captured from each animal at each 30 minute video recording. No images were selected if an animal was sleeping or grooming. Each image was subject to a blinding procedure by masking the area between the ears and cranial neck region (site of surgical incision) with a coloured rectangle (Microsoft Powerpoint for Mac 2011 v. 14.3.6, Microsoft Corporation, Redmond, WA, USA) to prevent distinguishing between pre- and post-operative images. Each image was scored for four facial features (“action units”, AU): orbital tightening, nose/cheek flattening, ear changes and whisker change [16]. Each AU was scored as “absent” (score = 0), “moderate”/“equivocal” (score = 1), or “obvious” (score = 2), and an average score (range 0–2) calculated.

Following image selection, images were randomised and scored independently by three blinded observers (VO, DDR, DSP) and the generated scores used to assess inter-rater reliability and analgesic intervention score generation. All images were scored by all raters, and all scoring was performed within a period of one week.

Prior to scoring the study images, a separate selection of approximately fifty images were scored by each observer and resultant scores discussed as a group as a training exercise in applying the RGS.

The second component required for analgesic intervention score generation is expert classification of images. Selection of experts was based on responses to a request to participate sent by electronic mail. None of the experts had previous experience with the RGS. Each expert was instructed to independently and blindly classify each image as either “pain” or “no pain”.

To test intra-rater reliability, the collected images were re-scored by the same observer (VO) one and six months after the initial scoring session.

Statistical analysis was performed with MedCalc v. 12.6.1.0 (MedCalc Software, Ostend, Belgium) and Graphpad Prism (GraphPad Prism v. 6.0b for Mac, GraphPad Software, La Jolla, CA, USA, www.graphpad.com). Internal consistency was assessed with Cronbach's alpha coefficient and standardized variables results reported for the overall scale and individual AUs (by recalculating alpha with each AU dropped). Inter- and intra-rater reliability, and agreement between experts, were assessed by calculating an intraclass correlation coefficient (ICC). An absolute model was used to calculate ICC and both single and average measures reported. The ICC was calculated for the complete scale and for each AU individually. Interpretation of the ICC was based on Altman (1991) and Landis and Koch (1977), with the following divisions: “very good” (0.81–1.0), “good” (0.61–0.80), “moderate” (0.41–0.60), “fair” (0.21–0.40), “poor” (<0.20) [25], [26]. Determination of an analgesic intervention score was performed by receiver operating characteristics (ROC) curve analysis. The ability of the RGS to discriminate between “pain” and no-pain” states was tested by comparing the area under the ROC curve (AUC) generated from image data with an AUC of 0.5. Unless stated otherwise, data are presented with 95% confidence intervals.

The RGS scores from the sham treatment group were tested with a one-way ANOVA for repeated measures followed by a Sidak post hoc test for differences from baseline. A p value<0.05 was considered significant.

To confirm the expected time course of post-operative pain, RGS scores between treatment groups were pooled and plotted.

Institutional ethics approval was provided by the Health Sciences Animal Care Committee, operating under the auspices of the Canadian Council on Animal Care (certificate no. AC11-0044).

## Results

Eighty-seven images were collected and scored from 16 different animals. No surgical complications such as infection or incision breakdown were observed in any animal. In cases where an action unit was not clearly discernible as a result of motion artefact or image quality, the number of action units used to calculate Cronbach's alpha and the ICC were reduced as follows: eyes (1/87 images not scored), ears (5/87 images not scored), nose (6/87 images not scored), whiskers (23/87 images not scored). Average RGS scores, from the full set of 87 images, were used for generation of an analgesic intervention score.

Five experts were recruited to participate in the study, with rat-specific experience ranging from 2–35 years and holding a variety of advanced training qualifications: registered laboratory animal technician (RLAT, n = 3), registered animal health technician (n = 1, also held RLAT), PhD and post-doctoral fellowship (n = 2). Inter-rater agreement between experts was very good (ICC average 0.82 [0.76–0.88]).

### Internal consistency

With the inclusion of all available AUs, internal consistency was 0.84 (0.78–0.90). The effect of dropping individual AUs was similarly influenced by ears (alpha = 0.75), eyes (alpha = 0.78) and nose (alpha = 0.78), and least affected by whiskers (alpha = 0.82), indicating that whiskers were the least useful AU contributing to the RGS.

### Inter-rater reliability

The single measure ICC was 0.85 (0.78–0.90) and the average measure ICC, reflective of multiple observers, resulted in greater reliability (ICC 0.94 [0.91–0.96], see Table 1). When each item was assessed independently whiskers were found to be the least reliable AU (as indicated from the internal consistency findings), with a single measure ICC of 0.52 (moderate), increasing to 0.76 (good) when multiple observers contributed to the score (Table 1).

**Table 1:** Inter-rater reliability with the Rat Grimace Scale.

Action unit	ICC single (95% CI)	ICC average (95% CI)
Orbital tightening	0.92 (0.89–0.95)	0.97 (0.96–0.98)
Ears	0.62 (0.51–0.72)	0.82 (0.76–0.88)
Nose/cheek	0.62 (0.51–0.72)	0.83 (0.75–0.89)
Whiskers	0.52 (0.39–0.63)	0.76 (0.66–0.84)
Average Action Units	0.85 (0.78–0.90)	0.94 (0.92–0.96)

Intraclass correlation coefficient (ICC) calculated for single and multiple (average) raters. The calculated ICC for individual action units (orbital tightening, ears, nose/cheek, whiskers) and for the average of all action units is shown.

Intra-rater reliability

Intra-rater reliability was very good with a single measure ICC of 0.83 (0.76–0.89) indicating that a single observer was able to reliably employ the RGS reliably over a period of time.

### Analgesic intervention score

**Figure 1:**
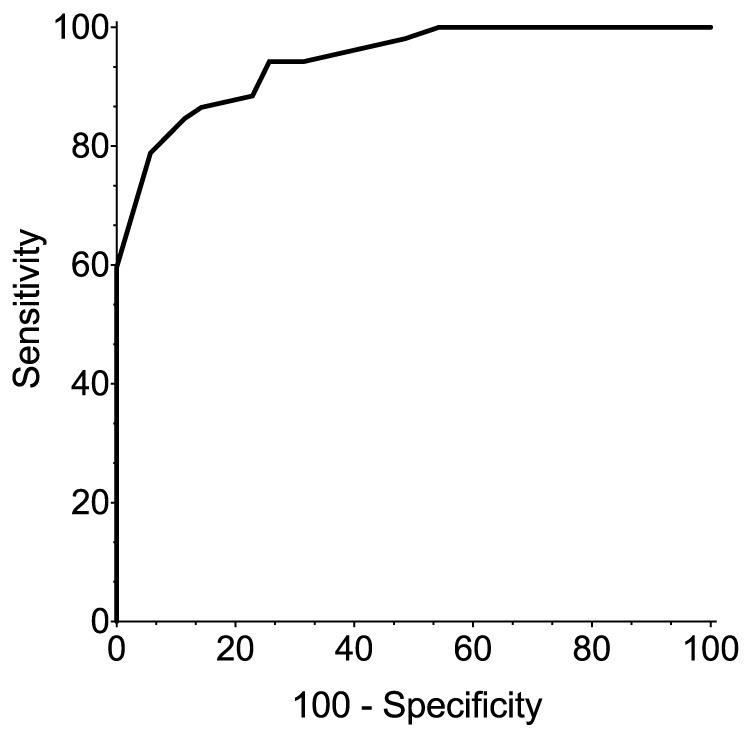
Receiver operating characteristics (ROC) curve. The optimal analgesic intervention score was >0.67, derived from a balance between the greatest values of sensitivity (84.6% [71.9–93.1]) and specificity (88.6% [73.3–96.8]. There was a significant difference in the area (0.94 [0.87–0.98], p<0.0001) under the calculated ROC curve compared with an AUC of 0.5 (representative of a non-discriminative test). n = 87 images

**Figure 2:**
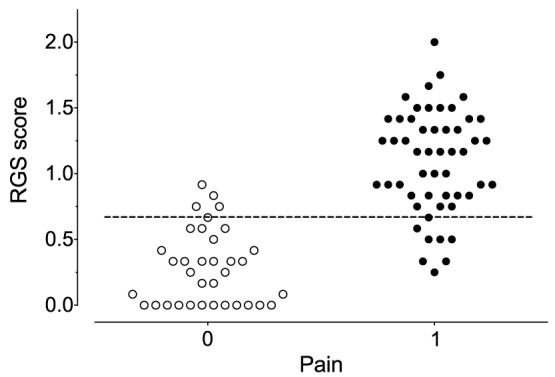
Scatter plot of Rat Grimace Scale scores categorised by “no pain” (0) and “pain” (1) assignment. Images of rat faces were scored by expert raters and classified as “pain” or “no pain” based on subjective evaluation. The dashed horizontal line shows the analgesic intervention score of 0.68. There is clear overlap between the two assigned pain states, reflective of the balance in sensitivity and specificity. n = 87 images

Experts classified 53 of the 87 images viewed as “pain”, with the remainder classified as “no pain”. The generated ROC curve (Figure 1) revealed the RGS to have an AUC of 0.94 (0.87–0.98), indicating it to be highly accurate (p<0.0001) for identification of a “pain” state [27]. An analgesic intervention score of >0.67 (out of a maximum total score of 2 from the RGS) was derived from the intersection between the greatest values of sensitivity (84.6% [71.9–93.1]) and specificity (84.6% [73.3–96.8], Figure 1 and Table 2). A scatter plot of the expert classified images in the context of the generated analgesic intervention score of >0.67 illustrates the potential for overlap when implementing an intervention score (Figure 2).

**Table 2:** Potential analgesic intervention scores with associated sensitivity and specificity values.

Intervention score	Sensitivity (%)	95% CI	Specificity (%)	95% CI
≥0	100.00	93.2–100.0	0.00	0.0–10.0
>0	100.00	93.2–100.0	34.29	19.1–52.2
>0.08	100.00	93.2–100.0	34.29	23.9–57.9
>0.17	100.00	93.2–100.0	45.71	28.8–63.4
>0.25	98.08	89.7–100.0	51.43	34.0–68.6
>0.33	94.23	84.1–98.8	68.57	50.7–83.1
>0.42	94.23	84.1–98.8	74.29	56.7–87.5
>0.5	88.46	76.6–95.6	77.14	59.9–89.6
>0.58	86.54	74.2–94.4	85.71	69.7–95.2
>0.67*	84.62	71.9–93.1	88.57	73.3–96.8
>0.75	78.85	65.3–88.9	94.29	80.8–99.3
>0.83	69.23	54.9–81.3	97.14	85.1–99.9
>0.92	59.62	45.1–73.0	100.00	90.0–100.0
>1	53.85	39.5–67.8	100.00	90.0–100.0
>1.17	44.23	30.5–58.7	100.00	90.0–100.0
>1.25	34.62	22.0–49.1	100.00	90.0–100.0
>1.33	26.92	15.6–41.0	100.00	90.0–100.0
>1.42	17.31	8.2–30.3	100.00	90.0–100.0
>1.5	9.62	3.2–21.0	100.00	90.0–100.0
>1.58	5.77	1.2–15.9	100.00	90.0–100.0
>1.67	3.85	0.5–13.2	100.00	90.0–100.0
>1.75	1.92	0.05–10.3	100.00	90.0–100.0
>2	0.00	0.0–6.8	100.00	90.0–100.0

An analgesic intervention score of >0.67 (*) was selected based on the balance of highest sensitivity (84.62%) and specificity (88.57%). 95% CI, 95% confidence intervals.

**Figure 3:**
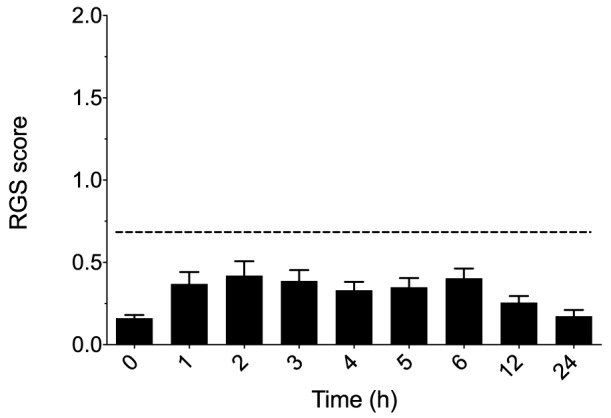
Sham treatment group and the Rat Grimace Scale. A significant overall effect of time was observed (p = 0.02) but differences between individual time points and baseline (time 0) were not significant. All RGS scores were below the analgesic intervention score of >0.67. Dashed horizontal line is set at RGS of 0.68. n = 9. Data are mean ± SEM.

In the sham treatment group (n = 9) there was a significant overall effect of time (p = 0.02) but differences were not identified when post-operative time points were compared with baseline (Fig. 3).

**Figure 4:**
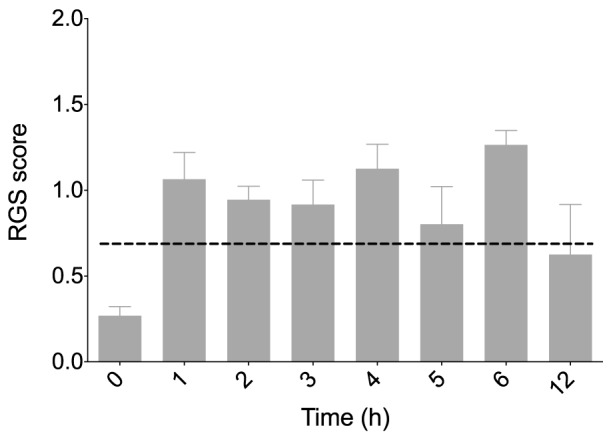
Time course of Rat Grimace Scale (RGS) scores. Pooled data (all treatment groups) show the expected increase in RGS post-operatively, followed by a decline at the 12 hour time point. [16] n = 16. Data are mean ± SEM.

The time course of post-operative RGS scores was as expected, with an increase in pain over the first 6 hours followed by a reduction by hour 12 (Fig. 4).

## Discussion

The purpose of this study was to assess the reliability of RGS scores and identify an analgesic intervention score. There are three main findings from this study: 1. the RGS exhibits excellent internal consistency, 2. inter- and intra-rater reliability of the recorded RGS scores is very good and 3. an analgesic intervention score has been generated successfully. These findings provide evidence for the utility of the RGS in a heterogenous setting (different experimental model, rater(s), environment), while generation of an analgesic intervention score expands the application of the RGS scale, moving it towards real-time assessment and application [23].

Evaluation of internal consistency, as measured with Cronbach's alpha, is affected by sample size and number of scale items. As a result it is somewhat arbitrary to assign classifications such as “good” or “excellent” to alpha values without reporting these factors. Ponterotto and Ruckdeschel (2007) proposed that values of alpha greater than 0.75 are excellent for scales with a low number of items (<7) and sample size less than 100 [28].

The scores and hence the reliability of an assessment tool such as the RGS is context and content specific and dependent on the characteristics of the setting including the rater(s), environment and patient/model [23]. The current study addresses the concerns raised by Sotocinal et al. (2011) and provides evidence of the utility of the tool in a heterogenous setting [16], [23]. Studies reporting the use of the RGS (or other assessment tools) should assess and report reliability. Eventually, this will allow reliability of RGS to be characterised in terms of expected reliability in a wide range of settings, and to what degree these factors influence reliability.

The presented data suggest that the AU whiskers score provides the lowest contribution to internal consistency and inter-rater reliability. This finding could reflect the difficulty in scoring whiskers as 23 of 87 images were not scored for this AU. It is possible that improvements in image quality through improved hardware or lighting conditions, or both, could positively affect the use of this AU. Video-camera capability has been shown to improve scoring accuracy, though is not a guarantee [15], [22]. Leach et al. (2012), despite using high-definition video-cameras, reported difficulties in scoring whiskers, and did not include this AU when using the MGS [22].

Inter-rater reliability data can be reported as either a value averaged over multiple raters or a value calculated for a single rater. A coefficient calculated as an average over multiple raters is useful for research purposes when a single animal is scored by multiple raters. However, when evaluation is limited to a single rater (as is often the case in a clinical setting when the number of available personnel is limited and a decision is time-sensitive) a single rater report is more representative of scale reliability. The very good single rater inter-rater reliability value of 0.85 suggests that the RGS may lend itself well to a clinical setting, where a single clinician (properly trained) can use it reliably. This is similar to the coefficient of 0.90 previously reported for the RGS (though it is unclear if this was a single or average observer coefficient) and MGS (the average observer coefficient was reported) [15], [16]. Our findings differed from those of Sotocinal et al. (2011) when assessing the ICC for individual AUs [16]. Our coefficients ranged from 0.52 (whiskers) to 0.93 (orbital tightening) in contrast to the 0.86 (nose/cheek flattening) to 0.96 (orbital tightening) of their study. Of the four AUs contributing to the total RGS, whiskers were the least reliable. This is compounded when scored by a single rater. When reliability was evaluated with multiple raters, the ICC for whiskers increased to “good” (Table 1). As discussed above, better reliability may be achieved with improved image quality.

Intra-rater reliability, where one rater assessed the same images one and six months after the first scoring session was very good (ICC of 0.83). No additional RGS scoring was performed by this rater during this time. This suggests that the application of the RGS and the resultant scores are consistent over time, however further work is needed to understand the interplay between training and medium to long term single observer reliability.

The identification of an analgesic intervention score broadens the application of the RGS, moving it from a useful, albeit largely observational tool, to one where analgesic intervention can be based on objective criteria [10]. The intervention score table provided (Table 2) can be used to make informed decisions regarding the intervention score to be applied to their work. For example, if avoidance of under-treating pain is paramount, a lower intervention score would be applied, accepting that some animals unlikely to be in pain receive analgesics (increased sensitivity at the expense of specificity). Factors to consider when setting an analgesic intervention score should include anticipated level of pain, side effect profile of analgesic agents and procedures, experimental and welfare impact of intervention. Several factors support the analgesic intervention score presented. Firstly, application of our suggested intervention score (>0.67) to data published by Sotocinal et al. (2011) correctly identifies time points at which significant peaks in RGS scores occurred in the models studied (intraplantar complete Freund's adjuvant [CFA], intra-articular kaolin/carrageenan and laparotomy) [16]. Furthermore, application of our intervention score to the effect of morphine administration on RGS (following intraplantar CFA) correctly identifies RGS scores from the negative control (saline, no analgesia) group [16]. Secondly, our pooled data (Fig 4) showed the expected time course of RGS scores; an increase following surgery followed by a decrease [16]. From the study design it was not possible to assess the efficacy of individual analgesic protocols, as the goal was to collect images to assess use of the RGS rather than test hypotheses on analgesic efficacy. For this reason statistical analysis of the data was not performed. Further work is required in this area. Finally, we solicited experts with a broad range of practical and training experience, including roles in decision making regarding analgesic intervention.

The sham treatment group controlled for any effects of anaesthesia or analgesia, or both, on the RGS. Though a significant overall effect of time was observed this did not persist following post hoc comparisons and RGS scores were less than the calculated analgesic intervention score of >0.67 in every case. A similar effect of anaesthesia has been reported in mice (given isoflurane alone) [10]. Further work is required to separate any effects of different anesthetic or analgesic protocols on the RGS.

### Limitations

Limitations of this study include the experimental model employed, lack of a negative (no analgesia) control group, reliance on expert assessment of pain behaviour in a non-verbal species, limitation to a single sex and the possibility of a training effect on inter-rater reliability.

This study was designed to further evaluate reliability of RGS scores in a heterogenous setting. Our choice of experimental model was incidental and though this limits a direct comparison with previous findings, it provides valuable additional information on the translation of the RGS between models [16]. This is supported by the apparent extrapolation of the analgesic intervention score presented here to the three inflammatory models used in development of the RGS [16]. Our model is similar in the type of pain created, but has the added advantage of mimicking a common procedure, rather than a standardised, well-controlled pain model. This finding strengthens the case that the RGS and the resultant scores may be applicable to other models of inflammatory pain.

We did not include a negative control group in this study as extreme cases on a measurement scale are not difficult to discriminate, and negative controls were included during initial construct validation [16].

The limitation of defining an analgesic intervention score based on expert judgement is the inherent subjectivity of the experts in assigning a “pain” - “no pain” classification to images. This limitation is unavoidable due to the absence of a gold standard measure of pain in rats, a problem shared with all non-verbal subjects. In such situations the use of expert judgement is an acceptable alternative [19], [24].

Our data are restricted to a single sex and strain, female Sprague-Dawley rats. No sex difference was identified in Wistars but lack of strain and sex differences in response to analgesia and exhibited pain behaviours should not be assumed [15], [16], [29].

The three blinded observers (VO, DDR, DSP) trained themselves in the application of the RGS prior to scoring study data. It is likely that there was a beneficial effect of training on scoring consistency, though it was not a goal of this study to investigate a training effect.

The practical utility of a measurement scale, such as the RGS, should extend beyond validity and reliability to also include training requirements, time to apply the scale and its complexity [24], [30]. Training in the use of the scale is yet to be formally assessed and tools are available to do this [31]. Complexity of the RGS is reduced by limiting the observation area to the face with a required focus on only 3–4 AUs (depending on whether whiskers are included in the scoring). It may be that human observers are particularly tuned to facial observations, raising the intriguing possibility that facial expression scales may be easier to learn [13]. A current limitation to real-time application is time to employ the scale and the retrospective nature of generated scores. While this has been dramatically improved through facial recognition software, facilitating partial automation (not used in this study), the goal should be real-time application [16]. This is a worthy aim as successful translation would truly expand the scope and impact of this tool.

The presented findings provide evidence of the utility of the RGS, with successful translation to a different experimental setting, model and raters. Important progress has been made towards development of a pain scale that spans research and clinical needs by identification of an analgesic intervention score.

## Supporting Information

Table S1 **The ARRIVE Guidelines Checklist.**
(PDF)
